# Examining Variability in Intra-Hospital Patient Referrals to Specialized Palliative Care: A Comprehensive Analysis of Disciplines and Mortality

**DOI:** 10.3390/jcm13092653

**Published:** 2024-04-30

**Authors:** Claudia Fischer, Katharina Dirschmid, Eva Katharina Masel

**Affiliations:** 1 Department of Health Economics, Center for Public Health, Medical University of Vienna, 1090 Vienna, Austria; n11825302@students.meduniwien.ac.at; 2 Department of Medicine I, Division of Palliative Medicine, Medical University of Vienna, 1090 Vienna, Austria; eva.katharina.masel@meduniwien.ac.at

**Keywords:** advance care planning, Austria, cancer, palliative care, specialized palliative care, supportive care, end-of-life care

## Abstract

**Background:** In Austria, specialized palliative care (SPC) access is limited, with unclear referral criteria, making it challenging to identify hospitalized patients requiring SPC and determine referral timing and mortality at the palliative care unit (PCU). **Methods:** This retrospective cohort study analyzed patients who underwent a palliative care (PC) needs assessment between March 2016 and November 2021 and were subsequently admitted to the PCU of Austria’s largest academic hospital. Demographic, clinical, and standardized referral form data were used for analysis, employing descriptive statistics and logistic regression. **Results:** Out of the 903 assessed patients, 19% were admitted to the PCU, primarily cancer patients (94.7%), with lung (19%) and breast cancer (13%) being most prevalent. Common referral reasons included pain (61%) and nutritional problems (46%). Despite no significant differences in referral times, most patients (78.4%) died in the PCU, with varying outcomes based on cancer type. Referral reasons like pain (OR = 2.3), nutritional problems (OR = 2.4), and end-of-life care (OR = 6.5) were significantly associated with the outcome PCU mortality. **Conclusions:** This study underscores Austria’s SPC access imbalance and emphasizes timely PC integration across disciplines for effective advance care planning and dignified end-of-life experiences in PCUs.

## 1. Introduction

Discussing goals of care with patients and making plans about where to receive care at the end of life (EOL) as well as addressing the preferred place of death are essential elements of advance care planning (ACP) [1]. As part of comprehensive palliative care (PC), ACP aims to enable seriously ill patients to participate in EOL decisions, which are crucial elements of person-centered care [2,3]. However, only a minimal number of patients die in their preferred place [4], which is usually their home [5,6]. For example, a study analyzing over two million death certificates and linked healthcare statistics from 14 countries showed that hospitals are still the predominant place of death (e.g., Hungary with 66.2% hospital deaths) [1]. This is also the case in Austria, where 50% of deaths currently occur in hospital [7]. However, a significant number of hospital admissions in the last months of life are potentially avoidable [8,9,10]. For certain patients in a PC setting and patients at the EOL, inpatient admission is justified because their condition requires monitoring and management in a hospital setting (e.g., because of the need for blood products or the development of complex infections) [11,12]. However, in contrast to acute care units, where providing high-quality EOL care is challenging [13,14], specialized hospital-based palliative care units (PCUs) have the resources to accommodate patients with complex EOL needs. In PCUs, specialized palliative care (SPC) doctors and nurses and other multidisciplinary team members (such as chaplains, social workers, and psychologists) [10] use a holistic approach to providing symptom management as well as psychosocial and spiritual support [15]. As resources for PC are scarce, it is crucial to provide access to those patients who are most in need [10].

Although several risk models (e.g., PiPS-A, PiPS-B) exist for mortality, their use in clinical practice is not recommended, and they are mostly used for research purposes [15]. While previous studies have examined patients admitted to SPC [16,17,18], there appears to be a lack of information about the intra-hospital allocation of patients to SPC via referrals from general wards. This information would, however, be crucial, especially for countries where there are limited standardized assignment criteria for SPC referrals, as is currently the case in Austria. For example, in a recent study, Gradalski and Kochan (2023) compared referral forms with the documentation of 300 patients referred to SPC and found that half of the referrals did not have sufficient information to justify prequalification on the basis of the documentation alone. As a result, the authors recommended that referral forms should be expanded to include a checklist of typical patients’ concerns [19].

Consequently, it remains unclear which patients and what kinds of PC and EOL care needs are prioritized in SPC referral decisions and which of the patients with complex needs are dying in PCUs providing SPC. While it is believed that cancer patients are the largest group receiving SPC, the specific types of cancer and group sizes being referred to SPC from general hospital units remain unknown. Furthermore, while in some countries, the number of hospital deaths has decreased significantly in recent years (e.g., in the United Kingdom from 68% (2009) to 44% (2019)), this is not the case in Austria, where the hospital death rate has remained quite constant since 2009 [7]. This raises the question as to whether the coordination of care for patients with complex needs can be improved, calling for greater insight into actual practice and the need to assess who is currently prioritized for SPC in Austria. Against this background, the aim of the current study was twofold. We aimed (i) to gain initial insights into the population of hospitalized patients referred to SPC from general hospital wards and (ii) to explore differences relating to referral time, reasons for referral assessed using a standardized referral form including a list of patient concerns, and PCU mortality.

## 2. Methods

This manuscript was drafted following the Strengthening the Reporting of Observational Studies in Epidemiology (STROBE) recommendations to assure reporting quality [20,21]. The STROBE reporting checklist is provided in the Table S1.

### 2.1. Study Design

We conducted a retrospective cohort study at the General Hospital of the City of Vienna (AKH). The AKH is also a university hospital and the site of the Medical University of Vienna. It is the fifth largest hospital in Europe, with approximately 1700 beds and more than 61,000 hospitalizations in 2021. We included data from more than five years (March 2016 to November 2021). The Department of Palliative Medicine, which provides SPC, includes a 12-bed PCU with approximately 300 admissions per year.

### 2.2. Patient Population

The study included adult patients (aged 18 years or older) who were referred to the PCU of the AKH following an assessment of PC needs by a palliative care consultation service (PCCS) that the patients underwent while being admitted to one of the general wards in the AKH. A PCCS is requested by a physician in a general ward when there is a need for PC or referral to the PCU. The information registered in the course of the PC needs assessment includes the patient’s name, diagnosis, and the reason(s) why conciliar care or referral may be required. For this purpose, the multiprofessional PC team at the AKH developed a PCCS assignment sheet based on the PERS(2)ON score, which includes common reasons for assignment to PC such as pain, eating, rehabilitation, social situation, suffering, dyspnea, and nausea. One reason for PC assignment worth mentioning separately is relief for family members. It is crucial in the context of respite care as it allows primary caregivers the opportunity to sustain their own well-being and continue providing optimal care. Respite care provides temporary relief too, allowing family members to take a break from their caring responsibilities, and therefore prevents burn-out. It can be arranged for different periods of time, ranging from hours to several days or even weeks [22].

### 2.3. Data Collection

Administrative data were reviewed to identify eligible patients during the study period. Data were abstracted from the electronic hospital database by two independent clinicians in training. To ensure high data quality, hand searches of patient records were also performed. This was conducted by searching for patients’ full names and dates of birth. This resulted in a complete dataset with no missing values. The variables gender (sex), age, referring unit, date of hospital admission, date of PCCS, and date of discharge were extracted directly from the electronic hospital database, as were diagnosis, reason for request, reason for referral, Eastern Cooperative Oncology Group (ECOG) performance status, date of referral to the PCU, place of discharge, and in-hospital mortality. Demographic information (i.e., gender, age) and clinical information, including “diagnosis” (in groups according to ICD-10 (10th International Classification of Diseases), were available for all patients. Information on the patient’s referral to the PCU included the following details: time between hospital admission and PCCS (in days), time between PCCS and admission to the PCU (referral time in days), length of stay in the PCU, the names of referring departments (grouped by medical specialty), and reasons for referral. Reasons for referral were assessed using a standardized referral form and included patient concerns such as care-giving difficulties, deterioration in general health, dyspnea, nutritional problems, psychological problems, nausea, vomiting, pain, relief for family members, social situation, EOL care, and other reasons such as rare refractory symptoms or complex wound management at the time of PCCS and/or around the time of admission to the PCU. This information was recorded in council and referral letters written by clinicians in the general wards as well as in inpatient records, electronic medical reports, and nurses’ protocols in the PCU. Information was also added on the patients’ level of functioning in terms of self-care, activities of daily living, and physical ability, measured by ECOG performance status, and the number of reasons for referral. ECOG performance status ranges from zero (no limitations) to five (death) [23]. The ECOG performance status of the patient population ranged from one to four and was therefore divided into four subgroups. The number of reasons for referral was calculated by adding up the reasons for referral for each individual patient, ranging from a minimum of one to a maximum of twelve reasons. This variable was grouped into four subgroups as for our patient population the score ranged from one to seven (i.e., 1, 2, 3, and 4–7). The primary outcome tested in this study was mortality in the PCU, defined as the patient’s place of discharge (death in the PCU or discharge).

### 2.4. Statistical Analysis

A descriptive analysis was performed to provide an overview of the patient population. Metric data were analyzed using mean, median, standard deviation (SD), and the interquartile range (IQR). Absolute and relative frequencies (percentages) were calculated for nominal and ordinal variables. We conducted tests for normality on all metric variables using Shapiro–Wilk tests. Differences in demographic or clinical variables between patients who died and those who were alive at discharge were estimated using appropriate statistical tests, including Mann–Whitney U, Chi^2^, Fisher’s exact, Fisher–Freeman–Halton exact tests, or *t*-tests as applicable.

Logistic regression analysis was used to examine the association between individual patient and referral characteristics and PCU mortality. Results were presented as odds ratio (OR) with a 95% confidence interval (95% CI) and the *p*-value. All statistical analyses were performed using IBM SPSS Statistics version 27.0.1. A two-sided significance level of alpha *p* < 0.05 was set for all statistical calculations.

### 2.5. Ethics

This study was approved by the Ethics Committee of the Medical University of Vienna (1333/2019; 1609/2022). Informed consent was not required as the study was observational in character, using administrative data rather than human tissue samples. Data were collected and stored in accordance with the provisions of the Austrian Data Protection Authority on the security of personal data. The study was conducted in accordance with the tenets of the Declaration of Helsinki.

## 3. Results

### 3.1. Description of Patient Population and Their Referrals to the PCU

A total of 903 patients received a PCCS in one of the general wards of the AKH during the observation period. Of these, 171 adult patients (19%) were referred to the PCU. Of the referred patients, 53.8% were female. The mean age at the time of PCCS was 59.6 years, with the youngest being 18 and the oldest 91 years old. The vast majority (94.7%) had malignancies such as lung cancer (18.7%), breast cancer (12.9%), or pancreatic cancer (10.5%). The remaining 5.3% had a non-malignant disease such as cystic fibrosis, chronic renal failure, or chronic ischemic heart disease. In this patient population, 49.1% of the patients had an ECOG score of two and 33.9% had a score of three, indicating that most of the patients were still able to care for themselves most of the time but needed assistance in some cases. Other baseline demographic and clinical characteristics of the patients admitted to the PCU are shown in Table 1.

Median referral times varied between the diagnosis groups; for example, the median referral time for bone/joint carcinoma or esophagus/gastric/small bowel and liver cancer was one day, whereas it was six days for patients with head and neck or pancreatic cancer (Table 2).

The majority of the patients (*n* = 141, 82.5%) received a PCCS due to a request for referral to the PCU, while the remaining 30 patients (17.5%) received a PCCS due to a request for conciliar care. Patients were referred to the PCU for single or multiple reasons. Pain was the predominant reason (61.4%), followed by nutritional problems (46.2%), a deterioration in general health (35.1%), and EOL care (30.4%). Other reasons included dyspnea, nausea, vomiting, psychological problems, social situation, relief for family members, and care-giving difficulties. The median length of stay in the PCU was 16 days (mean 23 days) and ranged from 1 to 203 days. The majority of the patients died in the PCU (78.4). Of the 21.6% of patients who were discharged, 18.7% were discharged to their home, 2.3% to a nursing home, and one (0.6%) person was referred to another department (Table 1).

### 3.2. Differences between Deceased and Discharged Patients

Patients who died in the PCU tended to be older compared to those who were discharged (deceased: mean (median) 61 (63) years vs. discharged: 56 (55) years) (*p* = 0.11), although this difference was not statistically significant. The median length of stay in the PCU was significantly shorter for patients who died in the PCU compared to those who were discharged (14 days vs. 23 days), indicating that patients who died were in a worse condition (*p* = 0.04). No significant differences were found in the time span between hospital admission and PCCS or in the referral time. When comparing patients who died with those who were discharged, differences in particular clusters of cancer were observed. For example, 20.9% of the patients who died in the PCU patients were living with lung/bronchial/mesothelial cancer, whereas in the group of the discharged patients, 10.8% had this diagnosis. Conversely, 6.7% of the deceased patients were living with colon/rectal/anal cancer, while only of the 16.2% discharged patients had this diagnosis. Regarding the referring department, only 8.2% of the deceased patients were referred by oncology departments compared to 16.2% of the discharged patients. There were also differences in the reasons for referral, with the following reasons being significantly more common in patients who died in the PCU than in those who were discharged: EOL care (*p* < 0.001), nutritional problems (*p* = 0.02), and pain (*p* = 0.03). The percentage of patients admitted for other reasons was significantly lower in deceased patients than in those who were discharged (*p* < 0.001). In addition, our analysis showed that the more referral reasons that were listed for a patient’s admission, the greater the likelihood that the patient would die in the PCU (*p* = 0.04) (Table 1).

### 3.3. Factors Associated with PCU Mortality

The following reasons for referral to the PCU were significantly and positively associated with death in the PCU: nutritional problems (OR = 2.44), pain (OR = 2.25), and EOL care (OR = 6.53). In addition, a sum of three referral reasons (reference category: one referral reason) was significantly associated with death in the PCU (OR = 4.00). In contrast, patients with a diagnosis of cancer of the colon, rectum, or anus (OR = 0.21, reference category: lung, bronchial, or mesothelial cancer) and patients who were referred due to infrequent refractory symptoms or complex wound management (OR = 0.22), for example, were more likely to be discharged from the PCU. Other independent parameters did not show significant differences in demographic and clinical characteristics between patients who died in the PCU and those who were discharged (Table 3).

## 4. Discussion

The aim of this study was to provide initial insights into the population of hospitalized patients who are referred to SPC, to investigate the timing of SPC requests, and to further investigate which of these patients die in the PCU in Austria. In summary, the results of the current study show that just over half of the patients who are referred to SPC in Austria are female (54%), that they predominantly have a cancer diagnosis (95%), and that their average age is less than 60 years. Patients who died in the PCU differed significantly from those who were discharged in terms of their diagnosis, the unit from which they were referred, their length of stay in the PCU, and the number and type of reasons for their referral. In addition, patients with the referral reasons “pain”, “nutritional problems”, and “EOL care” were significantly more likely to die in the PCU than patients without these referral reasons.

Our findings on the equivalence of in-hospital referrals to SPC in Austria are in line with previous studies from other countries. For example, a study conducted in Belgium analyzed cancer patients who died in PCUs [24]. In a multivariable logistic regression analysis comparing access to SPC for patients with solid cancer compared to those with hematologic cancer and considering socio-demographic characteristics, the study showed that patients who died of breast (OR = 2.25), respiratory (OR = 2.00), genitourinary (OR = 2.28), head and neck cancer (OR = 4.58), or other cancer types (OR = 2.60) were more likely to receive SPC than hematological cancer patients (reference category) (*p* = 0.02). Adsersen et al. (2017) also confirmed significantly lower chances of SPC admittance (*p* < 0.001) for patients with hematological malignancies such as leukemia (OR = 0.55) or non-Hodgkin lymphoma (OR = 0.57) [17]. In our study, only 3% of all patients referred to the PCU were diagnosed with hematological cancer. It can be concluded that patients with hematological cancer are not referred to the PCU in time or not at all [25,26].

While differences in the diagnosis of these patients may be an influencing factor, it is also known that individual judgements by clinicians as to whether someone is in the need of PC hinder equitable access [27]. The criteria for the exact timing of a referral to the PCU are heterogeneous and mostly depend on the policies of individual departments. This was also confirmed in the study by Vanbutsele et al. (2019) [24], in which physicians were asked about their perception of PC. In line with the low prevalence of patients with hematological cancers and the rather high prevalence of patients with breast cancer receiving SPC in our study, Vanbutsele et al. (2019) reported that 35.1% of physicians did not consider SPC to be meaningful (enough) for hematological cancer patients and therefore did not refer patients to it, while this was only the case for 5.3% for breast cancer patients [24]. Other studies have also concluded that PC is mostly seen as EOL care by hematological oncologists [28]. The distinction between primary and SPC competencies certainly requires a clear hospital policy and regular staff training. Rotations to the PCU may help improve the awareness of SPC competencies. In our study, we were able to gain important insights into reasons for referral, which were assessed using standardized referral forms, including patients referred mainly for social problems. These additional data, in combination with clinical information, provide valuable insights, especially considering the burden on SPC and the potential role of general home care, social support, and long-term care facilities [19].

For the future, standardized clinical decision support systems to increase awareness of the need for PC [15,29] and further studies focusing on the possible benefits of the timely integration of PC in oncology, particularly in hematological cancers, are needed. In view of high levels of in-hospital mortality, ACP, the definition of goals of care, and discussing preferred places of death appear to be mandatory to be able to decide which EOL care is the best for the individual patient [30]. The fact that approximately 80% of the patients who were referred to SPC died in the PCU illustrates that timely integration is not yet a reality in clinical practice. The delayed transfer of patients referred from hospital departments has also been observed in previous studies [17].

Our study showed differences in discharge probabilities across various tumor entities. Therefore, it seems important to study disease patterns to better predict the likelihood of spending EOL in hospital [31]. For example, patients with ovarian cancer or gastrointestinal stenosis may develop ileus at their EOL. Similarly, individuals with hematological disorders may require blood products or suffer from complicated infections. Beyond scrutinizing the impact of diverse diagnoses on PCU mortality, it is imperative to explore whether the disease stage could serve as a predictive marker for the timely implementation of palliative care and subsequent PCU mortality. Furthermore, the results of the current study underline that EOL care is basically provided in all units but that complex pain situations and EOL care not infrequently necessitate the need for SPC. In particular, patients referred to SPC for EOL and pain should be targeted for a discussion of ACP in clinical practice as they have a high likelihood of not being discharged from the PCU.

Our study has several limitations and strengths. Primarily, it was single-center and retrospective in design. The sample size was limited, and the analysis was confined to univariate analysis. The number of observations was limited and the analysis was restricted to univariate analysis. Certain variables that may have been associated with PCU deaths were not available in our dataset, such as APACHE IV [32]. On the other hand, our aim was to try and investigate associations with PCU deaths based on readily available and routinely collected data that can be used as an information base in clinical practice. What is more, our dataset had no missing data due to our additional data collection efforts. To the best of our knowledge, the present study is the first to be able to analyze access to SPC for hospitalized patients based on referral reasons collected using standardized referral forms that are not limited to a specific disease or specialty. In addition, when reporting the findings of our observational study, the latest reporting guidelines were followed [20,21]. Future research endeavors should aim to incorporate larger datasets from multiple centers to enable multivariate analysis, thereby providing a more comprehensive understanding of the factors influencing palliative care unit (PCU) mortality.

The complexity of defining a ‘good death’ underlines the need for EOL research so as to improve our understanding [33]. Austria and many other industrialized countries have a modular, tiered system of hospice and PC, ranging from basic to specialized facilities. It requires continuous research and outcome data in all of these areas. Regarding hospital settings, it is crucial to conduct larger prospective studies to determine how referral reasons influence access to SPC and the place of death. In addition, policy support is essential to improve EOL care, allocate resources, and promote integrative approaches rather than isolated silos [34].

## 5. Conclusions

Our study confirms that there is an imbalance in access to SPC in Austria, with certain patients more likely to receive SPC than others. Furthermore, significant differences in the likelihood of dying in the PCU can be observed, which calls for an increased awareness of the necessity of a timely integration of PC in all clinical areas. Reasons for referral can help identify those patients who are most likely to die in the PCU, allowing timely ACP and the greater likelihood of a ‘good death’ for such patients, as should be the goal for all other patients at their EOL as well.

## Figures and Tables

**Table 1:** Patient characteristics of total patient population and by patients who died/did not die in the palliative care unit.

Variables		Total Patient Population (*n* = 171)	Discharged Patients (*n* = 37)		Deceased Patients (*n* = 134)		
		*n* (%) or Mean (SD)/ Median (IQR)	(*n* (% of Discharged) or Mean/Median)		(*n* (% of Deceased) or Mean/Median)		*p*-Value
Age in years		59.58 (±14.73)/61 (21)	56.14 (±14.16)/55.00 (20)		60.54 (±14.80)/63.00 (21)		0.11 ^c^
	<50	41 (24.0)	11 (29.7)		30 (22.4)		
	50–60	43 (25.1)	12 (32.4)		31 (23.1)		
	61–70	39 (22.8)	5 (13.5)		34 (25.4)		
	>70	48 (28.1)	9 (24.3)		39 (29.1)		
Diagnosis groups							0.76 ^e^
	Bone/joint CA	5 (2.9)	2 (5.4)		3 (2.2)		
	Breast CA	22 (12.9)	5 (13.5)		17 (12.7)		
	Colon/rectal/anal CA	15 (8.8)	6 (16.2)		12 (6.7)		
	Esophagus/gastric/small bowel/liver CA	10 (5.8)	2 (5.4)		8 (6.0)		
	Head and neck CA	11 (6.4)	2 (5.4)		9 (6.7)		
	Hematological CA	5 (2.9)	1 (2.7)		4 (3.0)		
	Lung/bronchia/mesothelial CA	32 (18.7)	4 (10.8)		28 (20.9)		
	Other malignant diseases	15 (8.8)	2 (5.4)		13 (9.7)		
	Other non-malignant diseases	9 (5.3)	2 (5.4)		7 (5.2)		
	Ovarian CA	11 (6.4)	1 (2.7)		10 (7.5)		
	Pancreatic CA	18 (10.5)	6 (16.2)		12 (9.0)		
	Prostate/kidney CA	5 (2.9)	1 (2.7)		4 (3.0)		
	Skin CA	5 (2.9)	1 (2.7)		4 (3.0)		
	Vulva/vagina/uterus CA	8 (4.7)	2 (5.4)		6 (4.5)		
Gender (sex)							0.74 ^b^
	Female	92 (53.8)	19 (51.4)		73 (54.5)		
	Male	79 (46.2)	18 (48.6)		61 (45.5)		
Place of discharge							
	Death	134 (78.4)	0		134 (100.0)		
	Home	32 (18.7)	32 (86.5)		0		
	Nursing home	4 (2.3)	4 (10.8)		0		
	Referral	1 (0.6)	1 (2.7)		0		
Reason for referral to PCU			Yes	No	Yes	No	
	Care-giving difficulties	5 (2.9)	2 (5.4)	35 (94.6)	3 (2.2)	131 (97.8)	0.30 ^d^
	Deterioration in general health	60 (35.1)	10 (27.0)	27 (73.0)	50 (37.3)	84 (62.7)	0.32 ^b^
	Dyspnea	50 (29.2)	12 (32.4)	25 (67.6)	38 (28.4)	96 (71.6)	0.63 ^b^
	EOL care	52 (30.4)	3 (8.1)	34 (91.9)	49 (36.6)	85 (63.4)	**<0.001 ^b^**
	Nausea	24 (14.0)	6 (16.2)	31 (83.8)	18 (13.4)	116 (86.6)	0.67 ^b^
	Nutritional problems	79 (46.2)	11 (29.7)	26 (70.3)	68 (50.7)	66 (49.3)	**0.02 ^b^**
	Other	27 (15.8)	13 (35.1)	24 (64.9)	14 (10.4)	120 (89.6)	**<0.001 ^b^**
	Pain	105 (61.4)	17 (45.9)	20 (54.1)	88 (65.7)	46 (34.3)	**0.03 ^b^**
	Psychological problems	18 (10.5)	4 (10.8)	33 (89.2)	14 (10.4)	120 (89.6)	1.0 ^d^
	Relief for family members	7 (4.1)	2 (5.4)	35 (94.6)	5 (3.7)	129 (96.3)	0.65 ^d^
	Social situation	5 (2.9)	1 (2.7)	36 (97.3)	4 (3.0)	130 (97.0)	0.93 ^b^
	Vomiting	15 (8.8)	3 (8.1)	34 (91.9)	12 (9.0)	122 (91.0)	1.0 ^d^
Reason for request for SPC							0.46 ^b^
	Conciliary care	30 (17.5)	8 (21.6)		22 (16.4)		
	Referral	141 (82.5)	29 (78.4)		112 (83.6)		
Referring unit in groups							0.78 ^e^
	Cardiology/angiology/pulmonology	20 (11.7)	4 (10.8)		16 (11.9)		
	Dermatology	6 (3.5)	2 (5.4)		4 (3.0)		
	Endocrinology	7 (4.1)	1 (2.7)		6 (4.5)		
	Gastroenterology/hepatology	12 (7.0)	1 (2.7)		11 (8.2)		
	General Surgery	14 (8.2)	4 (10.8)		10 (7.5)		
	Gynecology	22 (12.9)	3 (8.1)		19 (14.2)		
	Hematology/hemostaseology	10 (5.8)	1 (2.7)		9 (6.7)		
	Infectiology	14 (8.2)	5 (13.5)		9 (6.7)		
	Nephrology/dialysis	9 (5.3)	1 (2.7)		8 (6.0)		
	Oncology	17 (9.9)	6 (16.2)		11 (8.2)		
	Orthopedics/trauma	10 (5.8)	3 (8.1)		7 (5.2)		
	Other	18 (10.5)	3 (8.1)		15 (11.2)		
	Radiation oncology	7 (4.1)	2 (5.4)		5 (3.7)		
	Rheumatology	5 (2.9)	1 (2.7)		4 (3.0)		
Time span between hospital admission and PCCS		13.74 (±22.08)/7 (13)	10.92 (±8.55)/7 (14)		14.51 (±24.51)/7.5 (13)		0.54 ^a^
	<4	46 (26.9)	6 (16.2)		40 (29.9)		
	4–7	41 (24.0)	14 (37.8)		27 (20.1)		
	8–16	43 (25.1)	7 (18.9)		36 (26.9)		
	>16	41 (24.0)	10 (27.0)		31 (23.1)		
Time span between PCCS and admission to PCU (referral time)		6.91 (±7.89)/4 (6)	6.76 (±9.52)/4 (6)		6.96 (±7.41)/4 (7)		0.56 ^a^
	<2	32 (18.7)	7 (18.9)		25 (18.7)		
	2–3	39 (22.8)	9 (24.3)		30 (22.4)		
	4–7	51 (29.8)	12 (32.4)		39 (29.1)		
	>7	49 (28.7)	9 (24.3)		40 (29.9)		
ECOG performance status							0.40 ^a^
	0	0					
	1	19 (11.1)	5 (13.5)		14 (10.4)		
	2	84 (49.1)	20 (54.1)		64 (47.8)		
	3	58 (33.9)	9 (24.3)		49 (36.6)		
	4	10 (5.8)	3 (8.1)		7 (5.2)		
	5	0					
Number of referral reasons							**0.04 ^a^**
	1	33 (19.3)	12 (32.4)		21 (15.7)		
	2	54 (31.6)	12 (32.4)		42 (31.3)		
	3	48 (28.1)	6 (16.2)		42 (31.3)		
	4	26 (15.2)	6 (16.2)		20 (14.9)		
	5	3 (1.8)	0		3 (2.2)		
	6	6 (3.5)	1 (2.7)		5 (3.7)		
	7	1 (0.6)	0		1 (0.7)		
Hospital mortality							
	Deceased	134 (78.4)					
	Discharged	37 (21.6)					
Length of PCU stay		22.59 (±24.07)/16 (23)	26.46 (±18.82)/23.00 (35)		21.52 (±25.29)/14.00 (21)		**0.04 ^a^**
	<8	46 (26.9)	7 (18.9)		39 (29.1)		
	8–16	42 (24.6)	6 (16.2)		36 (26.9)		
	16–30	41 (24.0)	11 (29.7)		30 (22.4)		
	>30	42 (24.6)	13 (35.1)		29 (21.6)		

^a^ Mann–Whitney U; ^b^ Chi^2^; ^c^ T-Test for independent samples; ^d^ Fisher’s exact test; ^e^ Fisher–Freeman–Halton exact test; CA: carcinoma; ECOG: Eastern Cooperative Oncology Group; EOL: end of life; IQR: interquartile range; PCCS: palliative care consultation service; PCU: palliative care unit; SD: standard deviation; SPC: specialized palliative care; Values in bold indicate statistically significant results.

**Table 2:** Referral times to palliative care unit.

Variables		Referral Time in Days (Time Span between PCCS and Admission to PCU)
		(Mean/Median)
Age in years		
	<50	7/4
	50–60	7/5
	61–70	6/5
	>70	7/4
Diagnosis groups		
	Bone/joint CA	5/1
	Breast CA	4/4
	Colon/rectal/anal CA	9/5
	Esophagus/gastric/small bowel/liver CA	4/1
	Head and neck CA	9/6
	Hematological CA	5/4
	Lung/bronchial/mesothelial CA	6/5
	Other malignant diseases	8/5
	Other non-malignant diseases	10/6
	Ovarian CA	7/4
	Pancreatic CA	12/5.5
	Prostate/kidney CA	3/2
	Skin CA	7/5
	Vulva/vagina/uterus CA	5/3
Gender (sex)		
	Female	6/4
	Male	7/4
Place of discharge		
	Death	7/4
	Home	7/4
	Nursing home	6/5.5
	Referral	4/4
Reason for request for SPC		
	Conciliary care	7/5
	Referral	7/4
Referring unit in groups		
	Cardiology/angiology/pulmonology	8/5.5
	Dermatology	7/6
	Endocrinology	9/7
	Gastroenterology/hepatology	13/6.5
	General surgery	7/5.5
	Gynecology	5/3.5
	Hematology/hemostaseology	5/3.5
	Infectiology	6/2
	Nephrology/dialysis	6/6
	Oncology	4/2
	Orthopedics/trauma	8/6
	Other	9/4
	Radiation oncology	7/6
	Rheumatology	6/4
Time span between hospital admission and PCCS		
	<4	6/5
	4–7	7/3
	8–16	6/4
	>16	10/6
ECOG performance status		
	0	
	1	8/4
	2	8/5
	3	5/4
	4	7/7.5
	5	
Number of referral reasons		
	1	6/4
	2	8/5.5
	3	8/4.5
	4	7/4.5
	5	1/1
	6	2/2
	7	4/4
Hospital mortality		
	Deceased	7/4
	Discharged	7/4
Length of PCU stay		
	<8	4/3
	8–16	8/4.5
	17–30	7/4
	>30	10/7

CA: carcinoma; ECOG: Eastern Cooperative Oncology Group; PCCS: palliative care consultation service; PCU: palliative care unit; SPC: specialized palliative care.

**Table 3:** Results of the univariable logistic regression analyses for death in the palliative care unit.

Variable		OR	95% CI Lower Bound	95% CI Upper Bound	*p*-Value
Age		1.02	1.00	1.05	0.11
Diagnosis groups					
	Lung/bronchia/mesothelial CA (reference)	-	-	-	0.83
	Bone/joint CA	0.21	0.03	1.70	0.15
	Breast CA	0.49	0.11	2.06	0.33
	Colon/rectal/anal CA	0.21	0.05	0.93	**0.04**
	Esophagus/gastric/small bowel/liver CA	0.57	0.09	3.71	0.56
	Head and neck CA	0.64	0.10	4.11	0.64
	Hematological CA	0.57	0.05	5.73	0.65
	Other malignant diseases	0.93	0.15	5.73	0.94
	Other non-malignant diseases	0.50	0.08	3.31	0.47
	Ovarian CA	1.43	0.14	14.35	0.76
	Pancreatic CA	0.29	0.07	1.20	0.09
	Prostate/kidney CA	0.57	0.05	6.48	0.65
	Skin CA	0.57	0.05	6.48	0.65
	Vulva/vaginal/uterus CA	0.43	0.06	2.90	0.39
Gender (sex)		1.13	0.55	2.35	0.74
Reasons for referral					
	Care-giving difficulties	0.40	0.06	2.49	0.33
	Deterioration in general health	1.61	0.72	3.60	0.25
	Dyspnea	0.83	0.38	1.81	0.63
	EOL care	6.53	1.91	22.39	**0.003**
	Nausea	0.80	0.29	2.19	0.67
	Nutritional problems	2.44	1.11	5.32	**0.03**
	Other	0.22	0.09	0.52	**<0.001**
	Pain	2.25	1.08	4.71	**0.03**
	Psychological problems	0.96	0.30	3.12	0.95
	Relief for family members	0.68	0.13	3.65	0.65
	Social situation	1.11	0.12	10.22	0.93
	Vomiting	1.12	0.30	4.18	0.87
Referring unit groups					
	Gynecology (reference)	-	-	-	0.82
	Cardiology/angiology/pulmonology	0.63	0.12	3.25	0.58
	Dermatology	1.74	0.16	18.80	0.65
	Endocrinology	1.27	0.11	14.05	0.85
	Gastroenterology/hepatology	0.63	0.05	7.75	0.72
	General surgery	0.39	0.06	2.27	0.28
	Hematology/hemostaseology	1.42	0.13	15.64	0.77
	Infectiology	0.29	0.06	1.46	0.13
	Nephrology/dialysis	0.79	0.14	4.49	0.79
	Oncology	0.29	0.06	1.40	0.12
	Orthopedics/trauma	0.32	0.04	3.55	0.28
	Other	0.40	0.05	3.04	0.37
	Radiation oncology	0.40	0.07	2.12	0.28
	Rheumatology	0.95	0.08	10.89	0.97
Time span between hospital admission and PCCS		1.01	0.99	1.04	0.39
Referral time		1.00	0.96	1.05	0.89
ECOG performance status					
	1 (reference)	-	-	-	0.55
	2	1.14	0.37	3.57	0.82
	3	1.94	0.56	6.75	0.30
	4	0.83	0.15	4.54	0.83
Number of referral reasons					
	1 (reference)	-	-	-	0.10
	2	2.00	0.77	5.20	0.16
	3	4.00	1.32	12.15	**0.01**
	4–7	2.37	0.80	7.03	0.12

CA: carcinoma; CI: confidence interval; ECOG: Eastern Cooperative Oncology Group; EOL: end of life; OR: odds ratio; PCCS: palliative care consultation service; Values in bold indicate statistically significant results.

## Data Availability

The datasets generated and/or analyzed during the current study are not publicly available due to organizational requirements for release but are available from the corresponding author upon reasonable request.

## Supplementary Materials

The following supporting information can be downloaded at: https://www.mdpi.com/article/10.3390/jcm13092653/s1, Table S1. The STROBE statement—Checklist of items that should be addressed in reports of observational studies.

## Abbreviations

ACP	Advance care planning
AKH	Allgemeines Krankenhaus der Stadt Wien (General Hospital of the City of Vienna)
CA	Carcinoma
CI	Confidence interval
ECOG	Eastern Cooperative Oncology Group
EOL	End of life
ICD-10	10th International Classification of Diseases
IQR	Interquartile range
OR	Odds ratio
PC	Palliative care
PCCS	Palliative care consultation service
PCU	Palliative care unit
PERS(2)ON score	P (pain) E (eating) R (rehabilitation) S (social situation, suffering) O2 (dyspnea) N (nausea/emesis)
PiPS A/B	Prognosis in Palliative care Study A/B
SD	Standard deviation
SPC	Specialized palliative care
STROBE	Strengthening the Reporting of Observational Studies in Epidemiology
